# Immunomodulatory Effects of Propolis on Endothelial Cytokine Release

**DOI:** 10.3390/molecules31122164

**Published:** 2026-06-19

**Authors:** Anna Kurek-Górecka, Małgorzata Kłósek, Grażyna Pietsz, Radosław Balwierz, Zenon P. Czuba

**Affiliations:** 1 Department of Microbiology and Immunology, Medical University of Silesia in Katowice, Jordana 19, 41-808 Zabrze, Poland; gpietsz@sum.edu.pl (G.P.); zczuba@sum.edu.pl (Z.P.C.); 2 Institute of Chemistry, University of Opole, Oleska 48, 45-052 Opole, Poland; radoslaw.balwierz@uni.opole.pl

**Keywords:** propolis, inflammation, fibrosis, cytokines, TGF-β, immune modulation

## Abstract

Propolis is a natural resinous product with a broad spectrum of biological activities, including immunomodulatory and anti-inflammatory effects. Increasing evidence suggests that propolis may influence pathways involved in tissue remodeling and fibrosis; however, comparative studies evaluating different propolis types in endothelial models remain limited. Brain microvascular endothelial cells, as a key component of the blood–brain barrier, constitute a relevant in vitro model for studying anti-inflammatory and neurovascular responses under both physiological and pathological conditions. The aim of this study was to compare the effects of Brazilian green propolis (EEP-BRA) and Polish brown propolis extracts (EEP-PL) on the immunological and fibrotic responses of brain microvascular endothelial cells. Human brain microvascular endothelial cells (hCMEC/D3-BBB) were exposed to propolis extracts (EEP-BRA and EEP-PL) under normoxic and hypoxic conditions to reflect diverse microenvironmental states. The analysis focused on the modulation of release of selected cytokines, including IL-10, IL-4, IL-6, IFN-γ, GM-CSF, TNF-α, IL-2, IL-8, and TGF-β, with particular emphasis on TGF-β as a key regulator of fibrosis. Results: Both propolis extracts significantly modulated cytokine production, although their effects differed depending on the origin of the propolis and oxygen conditions. Under the hypoxia condition followed by IFN-α stimulation, EEP-PL-50 was associated with reduced TNF-α (0.54 vs. 3.61 pg/mL; Hedges g = −6.78; large effect size, *p* > 0.05) and decreased TGF-β1, IL-8 and TGF-β2/β3. EEP-BRA-50 elicited a distinct profile characterized by increased IL-6 (171.58 vs. 27.63 pg/mL; *p* < 0.001; g = +6.15) and GM-CSF, while reducing TGF-β1. Both extracts preserved viability > 70% (ISO 10993-5). In conclusion, the results demonstrate that EEP-BRA and EEP-PL exert distinct immunomodulatory effects on brain endothelial cells. These findings highlight the importance of propolis origin in determining its biological activity and support its potential application in modulating inflammation and neurovascular responses.

## 1. Introduction

Propolis is a natural resinous substance produced by honey bees, characterized by remarkable biological activity that depends on its botanical and geographical origin. Honey bees collect resins from plant buds and bark and combine them with beeswax to form propolis, which they deposit within the nest to seal cracks and crevices, to lower the microbial load of the colony, to embalm intruders too large to be removed, and as a contribution to colony-level “social immunity” [1]. Among the most extensively studied types are Brazilian green propolis and European brown propolis of the Populus type, which differ markedly in their chemical composition; however, previous studies have demonstrated that both exhibit strong immunomodulatory and anti-inflammatory activity [2,3,4,5,6,7,8]. The biological activity of propolis results from its high polyphenol content. Typical components of European poplar-type propolis, which includes Polish propolis, are phenolic acids like cinnamic acid, caffeic acid, *p*-coumaric acid, and phenolic acid esters, especially caffeic acid phenethyl ester (CAPE), as well as flavonoids, especially chrysin, pinocembrin, galangin, hesperidin, naringenin, and quercetin [9,10]. Typical components of Brazilian green propolis are caffeoylquinic acid and prenylated cinnamic acid derivatives, such as artepillin C and baccharin [11].

Propolis exhibits potent immunomodulatory effects, acting as an anti-inflammatory agent that inhibits pro-inflammatory cytokines such as tumor necrosis factor-alpha (TNF-α), interleukin-6 (IL-6), interleukin-8 (IL-8), interleukin-1 beta (IL-1β), and granulocyte–macrophage colony-stimulating factor (GM-CSF) while modulating immune responses, often increasing the levels of anti-inflammatory mediators like interleukin-10 (IL-10). It regulates T cell proliferation and can enhance immune responses through interferon gamma (IFN-γ) and interleukin-4 (IL-4) under conditions of immunosuppression [8,12,13,14].

There is growing evidence that propolis may exert a significant regulatory effect on immune responses and inflammatory pathways that are closely associated with tissue remodeling and fibrotic processes. In particular, modulation of the cytokine network plays a key role in controlling chronic inflammation, endothelial dysfunction, and fibrosis. However, comparative studies on the immunomodulatory and endothelium-protective effects of different types of propolis on vascular endothelial cells are still limited. The endothelium of cerebral vessels is a key structural and functional component of the blood–brain barrier and is a very important in vitro model for studying the inflammatory and vasculoprotective effect in neurological and systemic diseases. Endothelial cells actively participate in immune signaling, cytokine production, and vascular permeability regulation, making them a suitable model for evaluating the biological effects of natural products.

In the present study, we investigated the effects of Brazilian green propolis (EEP-BRA) and Polish brown propolis (EEP-PL) extracts on brain microvascular endothelial cells commonly used in blood–brain barrier research. The study focused on their immunomodulatory, anti-inflammatory, and vasculoprotective effect under both normoxic and hypoxic conditions. We analyzed the modulation of selected cytokines, including IL-10, IL-4, IL-6, IFN-γ, GM-CSF, TNF-α, IL-2, IL-8, and transforming growth factor (TGF-β), with particular emphasis on TGF-β, which plays a key role in endothelial function and vascular homeostasis, contributing to vasculoprotective effects [15].

By using endothelial cells as a biological model, this study aims to elucidate the potential of propolis in modulating inflammatory and vasculoprotective effects in the central nervous system, at least in part through modulation of the TGF-β axis, and highlight its potential relevance in pathological states associated with BBB dysfunction. It should be noted that the present study was not intended to directly assess BBB integrity or permeability. Moreover, hypoxia- and interferon-α-related signaling pathways were not directly validated.

We hypothesize that Brazilian green propolis and Polish brown propolis extracts exert distinct but complementary immunomodulatory effects on brain endothelial cells, modulating pro-inflammatory and profibrotic signaling pathways. Specifically, we propose that the two extracts exert origin-dependent effects on cytokine production and that both downregulate TGF-β-mediated fibrotic responses under normoxic and hypoxic conditions, thereby contributing to improved endothelial homeostasis and protection against inflammation-driven tissue remodeling.

## 2. Results

The conducted study was designed to determine the impact of extracts of ethanolic extract of Polish propolis (EEP-PL) and ethanolic extract of Brazilian propolis (EEP-BRA) on anti-inflammatory cytokines such as IL-4, IL-10, and IFN-γ and pro-inflammatory cytokines such as TNF-α, IL-6, IL-2, and IL-8 as well as GM-CSF. Moreover, the effect of EEP-PL and EEP-BRA on the concentration of three isoforms of TGF-β was evaluated.

### 2.1. Effect of Propolis Extracts on hCMEC/D3 Cell Viability Under Normoxic and Hypoxic Conditions, with and Without IFN-α Stimulation

**Figure 1:**
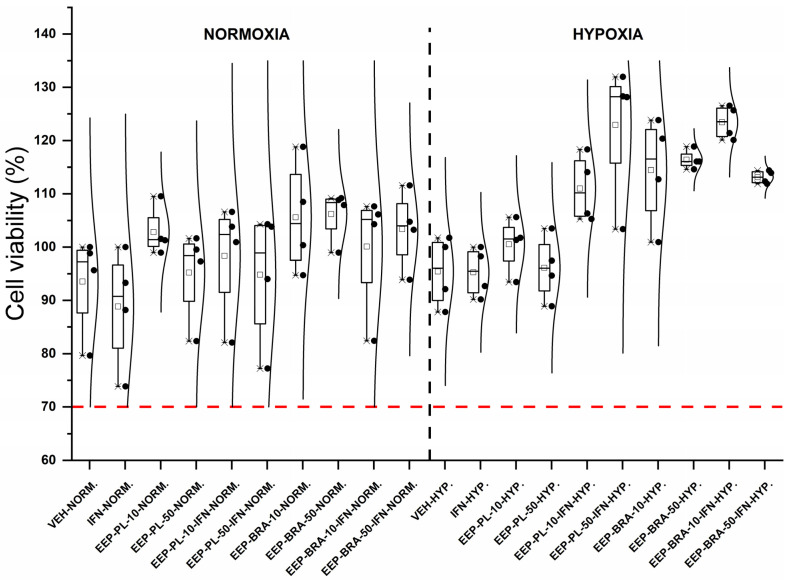
hCMEC/D3 cell viability evaluated by MTT (%)—the cytotoxic impact of propolis extracts (EEP-PL and EEP-BRA) in normoxic conditions, including IFN-α stimulation (IFN-NORM), and in hypoxic conditions, including IFN-α stimulation (IFN-HYP). The values represent the mean ± SD of four independent assays. In each box the central line denotes the median, the small square the arithmetic mean and the box the interquartile range. The whiskers extend to the minimum and maximum values, which are marked with the × symbol. The individual replicates (N = 4) are overlaid as points and the adjacent smooth curve depicts the fitted normal distribution of the values for that condition. The black vertical dashed line separates normoxic conditions (**left**) from hypoxic conditions (**right**) and the red horizontal dashed line marks the 70% cell viability threshold.

The viability of the hCMEC/D3 BBB cell line after the application of propolis extracts was determined using the 3-(4,5-dimethyl-2-thiazyl)-2,5-diphenyl-2H-tetrazolium (MTT) test. EEP-PL and EEP-BRA were used at two concentrations of 10 µg/mL and 50 µg/mL. The effect of EEP-PL and EEP-BRA on the metabolic activity of endothelial cells under normoxic and hypoxic conditions was evaluated and is depicted in Figure 1. The obtained results are presented in Table 1. ANOVA revealed a statistically significant effect of the experimental conditions on cell viability (F(19,60) = 5.84; *p* < 0.001).

According to the international standard ISO 10993-5:2009 for the biological evaluation of medical devices, a cell viability below 70% relative to the untreated control is determined to be indicative of cytotoxicity in the MTT assay [16].

In the control groups, no statistically significant differences in cell viability were observed between normoxic (VEH-NORM: 93.5 ± 9.4%) and hypoxic conditions (VEH-HYP: 95.4 ± 6.6%; *p* = 1.000). Similarly, IFN-α stimulation did not exert a cytotoxic effect. Although a decreasing trend in viability was noted under normoxia (88.8%), this difference did not reach statistical significance compared to the VEH control (*p* > 0.99).

These results indicate that the applied model of hypoxia combined with inflammatory stimulation does not induce cell death.

Under hypoxic and inflammatory conditions (IFN-α model), propolis extracts exhibited not only protective effects but also stimulation of mitochondrial activity (increase >100% relative to control). It was observed that EEP-BRA at a concentration of 10 µg/mL increased cell viability to 123.4%, representing a statistically significant difference compared to both the control (VEH-NORM, *p* < 0.001) and the inflammatory control (IFN-NORM, *p* < 0.001).

In contrast, EEP-PL required a higher concentration of 50 µg/mL (EEP-PL-50-IFN-HYP) to achieve a comparable effect (122.9%), showing statistical significance relative to VEH-NORM (*p* < 0.001) and IFN-NORM (*p* < 0.001).

Data are presented as mean ± standard deviation (SD), with a sample size of *n* = 4. Statistical analysis was performed using one-way analysis of variance (ANOVA), followed by Tukey’s HSD post hoc test for multiple comparisons.

### 2.2. Effect of Propolis Extracts on Cytokine Secretion Under Normoxic and Hypoxic Conditions, with and Without IFN-α Stimulation

**Figure 2:**
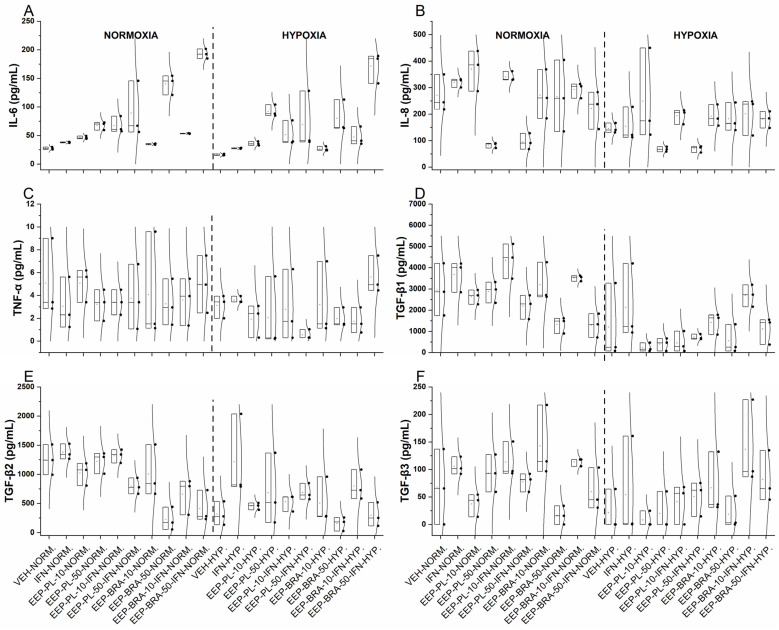

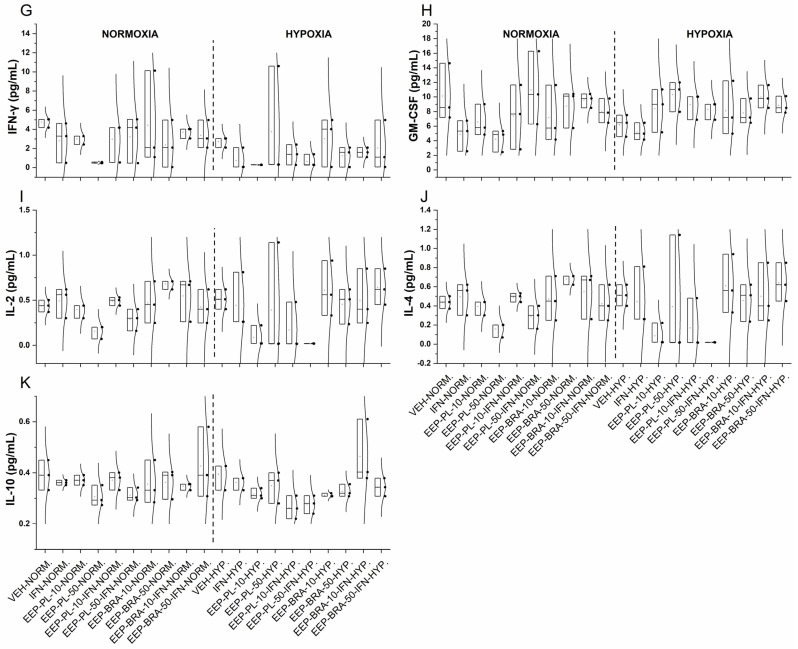
Concentrations of selected cytokines secreted by hCMEC/D3 cells under normoxic and hypoxic conditions, with and without IFN-α stimulation: (**A**) IL-6, (**B**) IL-8, (**C**) TNF-α, (**D**) TGF-β1, (**E**) TGF-β2, (**F**) TGF-β3, (**G**) IFN-γ, (**H**) GM-CSF, (**I**) IL-2, (**J**) IL-4 and (**K**) IL-10. Within each panel, the vertical dashed line separates normoxic conditions (left) from hypoxic conditions (right). In each panel, the box denotes the interquartile range, the central line the median, the whiskers the minimum–maximum range and the small square the arithmetic mean; the individual biological replicates (N = 3) are overlaid as points, and the smooth curve adjacent to each box depicts the fitted normal distribution of the values for that condition.

The concentrations of selected cytokines were measured under normoxic and hypoxic conditions following treatment with ethanolic extracts of Polish and Brazilian green propolis alone, as well as in combination with IFN-α. Both extracts increased IL-6 secretion under normoxia in a concentration- and origin-dependent manner: EEP-PL-50 (67.63 ± 7.09 vs. 27.55 ± 2.16 pg/mL for VEH-NORM; Tukey HSD *p* = 0.009; Hedges’ g = +6.11), EEP-BRA-50 (140.37 ± 17.25 pg/mL; *p* < 0.001; g = +7.34), and EEP-BRA-50+IFN-α (192.92 ± 8.32 pg/mL; *p* < 0.001; g = +21.76) (Figure 2A). Under hypoxic conditions, EEP-PL at concentrations of 10 and 50 μg/mL, and EEP-BRA at a concentration of 50 μg/mL (alone or in combination with IFN-α), significantly increased IL-6 levels to 92.48 ± 10.22 pg/mL and 171.58 ± 26.48 pg/mL, respectively (Figure 2A). In the case of IL-8 under normoxic conditions, it was observed that EEP-PL at a final concentration of 50 μg/mL alone and with IFN-α reduced IL-8 secretion both alone (83.14 ± 9.82 vs. 270.55 ± 69.77 pg/mL for VEH-NORM; Tukey HSD *p* = 0.004; Hedges’ g = −3.01) and in combination with IFN-α (95.50 ± 30.19 vs. 319.71 ± 16.91 pg/mL for IFN-NORM; Tukey *p* = 0.002; Hedges’ g = −7.33, 95% CI −12.66 to −2.00). The omnibus ANOVA on log10-IL-8 was highly significant (F(19,40) = 8.64, *p* < 0.001, η2 = 0.80) (Figure 2B). A pronounced reduction in TNF-α levels was observed under hypoxic conditions following stimulation of hCMEC/D3 cells with EEP-PL (50 μg/mL) in combination with IFN-α (Figure 2C). EEP-PL at a concentration of 50 μg/mL reduced TNF-α levels from 3.61 ± 0.29 to 0.54 ± 0.42 pg/mL. EEP-PL-50 co-administered with IFN-α was associated with a substantial reduction in TNF-α secretion under hypoxia (0.54 ± 0.42 vs. 3.61 ± 0.29 pg/mL for IFN-HYP control; mean difference −3.06 pg/mL, 95% CI −3.93 to −2.20; Hedges’ g = −6.78, 95% CI −11.79 to −1.83). The very large effect size with non-overlapping 95% CI of the mean difference indicates a robust biological signal; however, the omnibus one-way ANOVA on log10-TNF-α did not reach significance (F(19,40) = 1.67, *p* = 0.086, η2 = 0.44). Welch’s ANOVA on the same log-transformed data (Statistica 13.3, Robust Tests of Equality of Means) similarly did not reach significance (F(19,14.4) = 1.65, *p* = 0.170). Nevertheless, three-way factorial ANOVA on log10-TNF-α confirmed a significant main effect of oxygenation (*p* = 0.005) (Supplementary Table S3). EEP-PL at a lower concentration of 10 μg/mL reduced TNF-α levels to 2.77 pg/mL compared with the IFN-α control group. EEP-BRA showed a weak effect on TNF-α under hypoxic conditions. EEP-BRA, following IFN-α stimulation at a concentration of 10 µg/mL, reduced TNF-α levels to 1.74 ± 1.11, whereas at a concentration of 50 µg/mL, these levels rose to 5.62 ± 1.63. 

In addition to evaluating the effects of propolis extracts on pro- and anti-inflammatory cytokines, an analysis of their influence on three TGF-β isoforms (TGF-β1, TGF-β2, TGF-β3) was also performed. Reduced secretion of all three TGF-β isoforms was observed under hypoxic conditions. EEP-PL-50 + IFN-α most strongly inhibited the secretion of the profibrotic factor TGF-β1, reducing its level nearly threefold compared with the control group (728 vs. 2124 pg/mL; *p* < 0.001) (728.6 ± 126.9 vs. 2124.6 ± 1803.0 pg/mL); mean difference −1396 pg/mL; Hedges’ g = −0.87, 95% CI −2.58 to +0.84; one-way ANOVA on log10 omnibus F(19,40) = 5.14, *p* < 0.001, η2 = 0.71; Tukey HSD-adjusted *p* = 0.997, reflecting high within-group variance of the IFN-HYP control [SD = 1803, CV ≈ 85%]; three-way ANOVA confirmed oxygenation [*p* < 0.001], stimulation [*p* = 0.013] and treatment [*p* = 0.004] main effects), as shown in Figure 2D, suggesting a strong protective potential against tissue remodeling. EEP-BRA+IFN-α at a concentration of 50 µg/mL also significantly decreased the TGF-β1 level (1114.2 pg/mL; *p* < 0.001 vs. control) (1114.2 ± 641.1 pg/mL vs. 2124.6 ± 1803.0 pg/mL for IFN-HYP; mean difference −1010 pg/mL; Hedges’ g = −0.60, 95% CI −2.25 to +1.06; Tukey HSD-adjusted *p* = 1.00; three-way ANOVA, treatment main effect *p* = 0.004), as shown in Figure 2D. Analysis of the TGF-β2 isoform demonstrated that EEP-BRA most strongly reduced its concentration under hypoxic conditions at a concentration of 50 µg/mL, both alone and following IFN-α stimulation (155.9 vs. 315.6 pg/mL for VEH-HYP control (Tukey HSD *p* = 0.911 due to high control variance); 295.0 vs. 1214.0 pg/mL for IFN-HYP (Tukey *p* = 0.219); omnibus ANOVA on log10-TGF-β2 F(19,40) = 4.60, *p* < 0.001, η2 = 0.69; three-way ANOVA confirmed oxygenation [*p* < 0.001], stimulation [*p* = 0.023] and treatment [*p* < 0.001] main effects), as shown in Figure 2E. Moreover, under normoxic conditions, a statistically significant reduction in TGF-β2 concentration was also observed at the concentration of 50 µg/mL EEP-BRA (221.7 ± 198.0 vs. 1249.1 ± 258.9 pg/mL; mean difference −1027 pg/mL, 95% CI −1564 to −491; Hedges’ g = −3.57, 95% CI −6.51 to −0.62; Tukey HSD-adjusted *p* = 0.013), as shown in Figure 2E. In the case of isoform TGF-β3, it was noticed that similar to in the case of isoform TGF-β2 under normoxic conditions, EEP-BRA at a concentration of 50 µg/mL most strongly reduced its concentration (16.6 ± 16.9 vs. 67.5 ± 68.5 pg/mL; Tukey HSD *p* = 1.00; omnibus F(19,40) = 2.10, *p* = 0.024), as shown in Figure 2F. Under hypoxic conditions, EEP-BRA at a concentration of 50 µg/mL significantly decreased the TGF-β3 concentration from 37.1 to 29.1 pg/mL (Tukey HSD *p* = 1.00; three-way ANOVA, oxygenation *p* = 0.003, stimulation *p* = 0.004), as shown in Figure 2F.

It has been shown that EEP-PL, at a final concentration of 50 μg/mL, reduced IFN-γ levels under normoxic conditions, although this effect was not statistically significant (Figure 2G). In the case of GM-CSF, similarly to IL-2, IFN-α stimulation did not induce an increase in the secretion of granulocyte–macrophage colony-stimulating factor (Figure 2H). Therefore, the effects of both types of propolis on this protein are difficult to clearly interpret. Moreover, the obtained results did not reach statistical significance. An analysis of the IL-2 results showed that there was no increase in the secretion of this cytokine in the hCMEC/D3 cell line stimulated with IFN-α. Moreover, the obtained results were not statistically significant. Therefore, the effect of EEP-PL and EEP-BRA on IL-2 secretion remains inconclusive and does not allow for a clear interpretation (Figure 2I). Considering the effect of propolis on IL-4 concentration, it was observed that EEP-PL at 50 μg/mL alone caused an increase in concentration of IL-4 to 0.39 ± 0.65 pg/mL and at 50 μg/mL with IFN-α decreased the concentration of IL-4 to 0.017 ± 0.001 pg/mL (note: at or below the assay’s LLOQ) in hypoxic conditions (Figure 2J). Very low levels of cytokine secretion were observed for IL-10 in the cells in both normoxic and hypoxic conditions (Figure 2K). In the case of this cytokine, no statistically significant differences were observed.

### 2.3. Comparative Effects of EEP-PL and EEP-BRA on Selected Cytokines’ Secretion in IFN-α Induced by Human Brain Microvascular Endothelial Cells in Normoxic and Hypoxic Conditions

**Figure 3:**
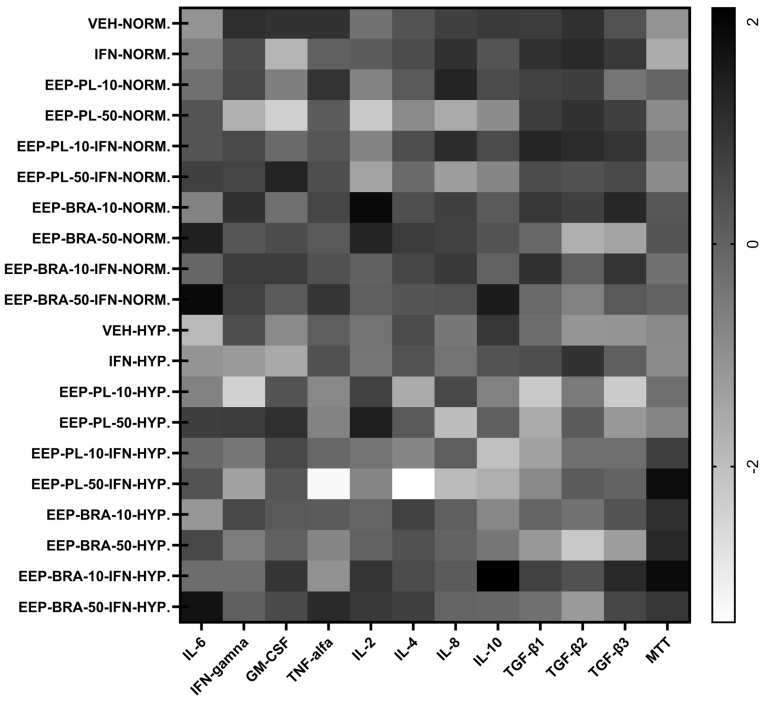
The heatmap shows the distribution of cytokine concentrations under normoxic and hypoxic conditions. Lighter areas correspond to lower cytokine concentrations, whilst darker areas indicate higher concentrations. Under hypoxic conditions, a general decrease in signal intensity is observed for several cytokines. In contrast, when exposed to ethanol extracts of propolis, signal intensity is variable, suggesting a potential immunomodulatory and anti-inflammatory effect.

In the lower part of the heatmap (hypoxic conditions), several pro-inflammatory cytokines appear lighter in color compared to the upper part, suggesting that the activity of pro-inflammatory cytokines is suppressed under hypoxic conditions and that ethanol propolis extracts have a specific effect under these conditions (Figure 3). EEP-PL at a concentration of 50 μg/mL with IFN-α in hypoxic conditions produced very bright patches for TNF-α and IL-2. At high concentrations, EEP-PL exhibits a strong anti-inflammatory effect under hypoxic conditions, markedly reducing TNF-α levels below those of the control group. EEP-BRA exhibits a more heterogeneous pattern. Under normoxic conditions (upper part of the heatmap) with EEP-BRA at a concentration of 50 µg/mL, darker regions are observed, which may indicate stronger stimulation of the immune system (i.e., an immunostimulatory effect) compared to EEP-PL.

**Figure 4:**
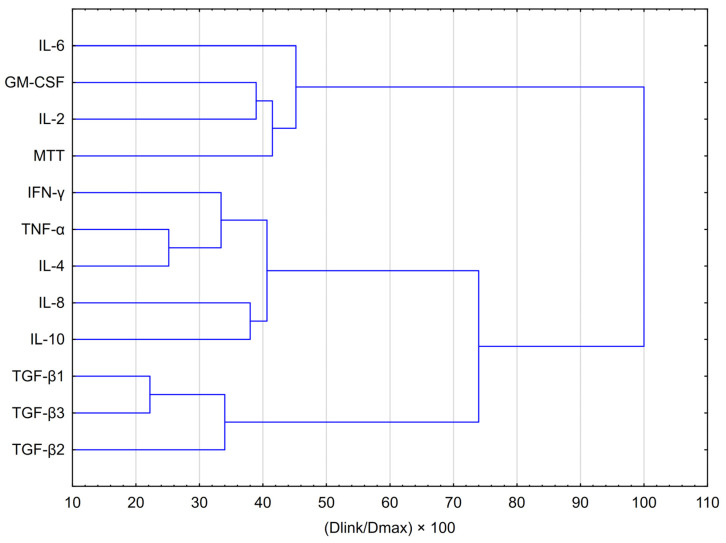
Dendrogram obtained by hierarchical cluster analysis (HCA) of cytokine secretion (IL-10, IL-4, IL-6, IFN-γ, GM-CSF, TNF-α, IL-2, IL-8, TGF-β1, TGF-β2, and TGF-β3) in hCMEC/D3 BBB cells stimulated with IFN-α and treated with EEP-PL or EEP-BRA under normoxic and hypoxic conditions. Dlink represents the linkage distance (i.e., the distance between clusters), while Dmax indicates the maximum distance between any clusters.

HCA (Figure 4) showed three clusters. The first cluster comprises IL-2, GM-CSF, and IL-6. The second cluster includes IFN-γ, TNF-α, IL-4, IL-8, and IL-10, representing a mixture of pro-inflammatory (e.g., TNF-α) and anti-inflammatory (e.g., IL-4, IL-10) markers. Their close proximity suggests that the investigated extracts (EEP-PL and EEP_BRA) modulate the inflammatory response in a comprehensive rather than selective manner. The third cluster consists of TGF-β1, TGF-β2, and TGF-β3. This forms a tight and distinct cluster (at a low Dlink/Dmax value), serving as a strong internal validation. This observation supports the precision of the assay, as these isoforms are biologically similar and subject to similar regulatory mechanisms.

**Figure 5:**
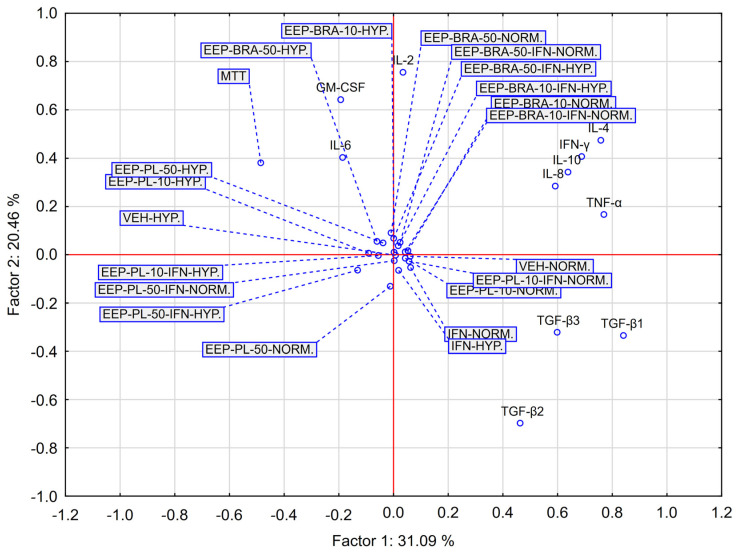
PCA score plot illustrating the impact of EEP-PL and EEP-BRA (10–50 μg/mL) on cytokine secretion (IL-10, IL-4, IL-6, IFN-γ, GM-CSF, TNF-α, IL-2, IL-8, TGF-β1, TGF-β2, and TGF-β3) by human brain microvascular endothelial (hCMEC/D3 BBB) cells stimulated with IFN-α. Abbreviation: PC, principal component.

To evaluate the overall variance in the experimental data and to determine whether the geographical origin of propolis (EEP-BRA vs. EEP-PL) differentially modulates cellular responses under normoxic and hypoxic conditions, principal component analysis (PCA) was performed (Figure 5).

The model extracted two principal components (PCs) with eigenvalues > 1, explaining 51.56% of the total variance (R^2^_{cum} = 0.516; cross-validated Q^2^_{cum} = 0.23). The first component (PC1), accounting for 31.10% of the variance, separated samples based on an inverse relationship between cellular viability and inflammatory activation. The second component (PC2), explaining 20.46% of the variance, revealed a separation of immunomodulatory profiles associated with propolis origin. Samples treated with EEP-BRA clustered in the upper quadrants and were positively associated with GM-CSF, IL-6, and IL-2. Notably, the TGF-β2 vector showed a distinct loading pattern (negative PC2) compared to other growth factors, suggesting involvement of a specific regulatory pathway. Overall, PCA indicates that both extracts modulate the immune response; however, the cytokine release profile depends on the geographic origin of the EEP, with EEP-BRA showing a stronger association with the GM-CSF/IL-6 axis.

## 3. Discussion

The blood–brain barrier (BBB) is a highly specialized physical barrier located between the bloodstream and the central nervous system. Its principal structural components include brain microvascular endothelial cells, astrocytes, and pericytes [17]. The capillary endothelium forming the BBB plays a crucial role in regulating cerebral blood flow and pressure, secreting biologically active factors, controlling the exchange of substances across the vessel wall, and participating in angiogenesis as well as inflammatory and immune responses [18]. Under pathological conditions associated with the release of pro-inflammatory cytokines, tight junctions between endothelial cells may become disrupted, leading to increased BBB permeability. Among these cytokines, TNF-α is of particular importance, as it induces the expression of other pro-inflammatory mediators, including IL-1β and IL-6. An in vitro study conducted on cultured brain microvascular endothelial cells (BMECs) demonstrated that TNF-α directly binds to its receptors, leading to increased permeability of the blood–brain barrier [19]. Both TNF receptors are required for direct TNF-mediated cytotoxicity in microvascular endothelial cells [20]. Exposure to TNF-α was associated with decreased expression of tight-junction proteins, including Zonula Occludens-1 (ZO-1), claudin-5, and occludin. The reduction in these proteins is mediated by multiple intracellular signaling pathways. In particular, TNF-α was shown to suppress claudin-5 promoter activity and downregulate its mRNA expression through activation of the nuclear factor kappa-light-chain-enhancer of activated B cells (NF-κB) signaling pathway [21]. Propolis and its bioactive components, such as flavonoids, have been reported to exert protective effects on the BBB, largely due to their strong antioxidant properties. These compounds are capable of scavenging reactive oxygen species and mitigating oxidative stress-induced cellular damage [22]. In the present study, a decrease in TNF-α concentration was observed under hypoxic conditions following stimulation of hCMEC/D3 cells with an ethanolic extract of Polish propolis (50 μg/mL) in combination with IFN-α; however, this change was not statistically significant. The parallel decrease in TNF-α and TGF-β1 concentrations may indicate that propolis attenuates pathways associated with inflammatory tissue remodeling and fibroproliferative responses. Immunomodulatory effects of Brazilian green propolis were reported by Machado et al., who demonstrated that propolis treatment significantly modulated cytokine production in an LPS-induced pulmonary inflammation model by reducing pro-inflammatory mediators while regulating anti-inflammatory cytokine responses [11]. However, in our study, EEP-BRA did not cause a strong inhibitory effect on the concentration of TNF-α under hypoxia compared to EEP-PL (Figure 2C). Studies conducted by other researchers demonstrated that propolis reduced the levels of IL-6 and TNF-α, exerting anti-inflammatory effects through the modulation of additional mediators, including NF-κB and IL-1β [23,24,25]. In contrast, a statistically significant increase in IL-6 levels was detected after treatment with Brazilian and Polish ethanol extracts of propolis in human brain microvascular endothelial cells under both normoxic and hypoxic conditions. Brain endothelial cells forming the BBB are highly sensitive to inflammatory stimulations and actively contribute to the immune response through the production of cytokines, including IL-6. Interleukin-6 plays an important role in regulating BBB permeability, among others by modulating the expression of tight-junction proteins [19,26]. IL-6 is a pleiotropic cytokine and can also act in both autocrine and paracrine manners on endothelial cells [27]. Its effects depend on multiple factors, including concentration, duration of exposure, and the presence of other cytokines. IL-6 may exert anti-inflammatory effects by decreasing the release of other pro-inflammatory cytokines [28].

Importantly, the observed increase in IL-6 levels does not necessarily indicate a harmful effect. A growing body of evidence suggests that IL-6 may also play a regulatory role, contributing to the adaptive response of the BBB to environmental and inflammatory stimulations. Further investigation into the molecular mechanisms underlying IL-6 induction is also warranted, particularly with respect to signaling pathways such as NF-κB, Signal Transducer and Activator of Transcription 3 (STAT3), and mitogen-activated protein kinases (MAPKs). Dose–response and time-course studies would help clarify whether the observed effects represent transient adaptive responses or sustained pro-inflammatory activation. Czpakowska J. et al. examined the effect of polyphenols, like curcumin, chrysin, myricetin and resveratrol, on concentrations of the pro-inflammatory cytokines IL-1β, IL-6, and IL-8 secreted by a cerebral microvascular endothelial cell line (HBEC-5i) stimulated with TNF-α. They have shown that myricetin and chrysin showed the strongest effect and decreased the concentrations of the pro-inflammatory cytokines secreted by brain endothelial cells [28]. A study conducted by Zamarrenho L. G et al. showed that a polar Brazilian propolis fraction increased IL-6 and IL-10 concentration in a macrophage model [29]. Treatment with chrysin and myricetin resulted in a significant decrease in IL-8 concentration, whereas resveratrol and curcumin demonstrated only a non-significant trend toward reduced inflammatory activity. In our studies, we observed that ethanolic extract of Polish propolis at a concentration of 50 μg/mL significantly reduces the concentration of IL-8 secreted by cerebral endothelial cells. Cheng X. et al. examined the effects of kaempferol on neuroinflammation and blood–brain barrier (BBB) damage caused by LPS in mice [30]. In the study, BALB/c mice received kaempferol (25, 50, or 100 mg/kg) for seven days before LPS was administered to induce inflammation. The study showed that kaempferol lowered the levels of inflammatory mediators such as IL-1β, IL-6, TNF-α, monocyte chemoattractant protein-1 (MCP-1), cyclooxygenase-2 (COX-2), and inducible nitric oxide synthase (iNOS) in brain tissue. It also helped preserve BBB integrity by increasing the expression of barrier-associated proteins, including occludin-1, claudin-1, and CX43. Additionally, kaempferol reduced high-mobility group box 1 protein (HMGB1) concentrations and inhibited the Toll-Like Receptor 4/Myeloid differentiation primary response 88 (TLR4/MyD88) signaling pathway at both the gene and protein expression levels.

The present study provides important insights into the cytotoxic and cytoprotective effects of Polish and Brazilian propolis extracts on brain endothelial cells under normoxic and hypoxic inflammatory conditions. Notably, the obtained results indicate that neither extract exerted overt cytotoxicity within the tested concentration range, confirming their relative safety toward endothelial cells under basal conditions. This observation is consistent with previous reports highlighting the biocompatibility of propolis-derived polyphenols in vascular models.

A key finding of this study is that hypoxic conditions appear to sensitize brain endothelial cells to the biological activity of polyphenolic compounds. While the effects of the extracts were moderate under normoxia, exposure to hypoxia combined with inflammatory stimulation markedly enhanced the cellular response. This phenomenon may be explained by metabolic reprogramming and increased oxidative stress under hypoxia, which can alter redox-sensitive signaling pathways and mitochondrial function, thereby increasing cellular susceptibility to exogenous bioactive compounds [31,32,33,34,35].

Importantly, both propolis extracts were able to counteract the metabolic impairment induced by hypoxic and inflammatory stress, as evidenced by the restoration—and even enhancement—of mitochondrial activity. This suggests a potential role of propolis-derived compounds in supporting endothelial cell metabolism under pathophysiological conditions associated with ischemia and inflammation. The observed increase in metabolic activity above control levels may reflect improved mitochondrial efficiency or activation of adaptive survival pathways.

Comparative analysis revealed a clear difference in potency between the two extracts. Brazilian propolis demonstrated a stronger biological effect, achieving comparable or superior enhancement of cell viability at a lower concentration than Polish propolis. This disparity may be attributed to differences in chemical composition, particularly the higher content of prenylated phenolic compounds such as artepillin C in Brazilian propolis, which are known for their pronounced bioactivity, including antioxidant and mitochondrial-modulating properties [36,37,38].

Taken together, these findings suggest that the biological effects of propolis are context-dependent and significantly influenced by the cellular microenvironment. The enhanced responsiveness of endothelial cells under hypoxic conditions highlights the potential therapeutic relevance of propolis in disorders involving vascular dysfunction, hypoxia, and inflammation, such as ischemic stroke or neurodegenerative diseases. Further studies are warranted to elucidate the precise molecular mechanisms underlying these effects and to determine the contribution of individual polyphenolic constituents.

Transforming growth factor-beta (TGF-β) comprises three main isoforms (TGF-β1, TGF-β2, and TGF-β3) and is a pleiotropic cytokine that plays a key role in the regulation of cellular processes, including proliferation, apoptosis, differentiation, cell migration, and the synthesis of extracellular matrix components.

Particular importance is attributed to TGF-β due to its involvement in modulating inflammatory responses and initiating fibrotic processes. This cytokine promotes fibroblast activation, their differentiation into myofibroblasts, and the enhanced production of collagen and other structural proteins, thereby contributing to tissue remodeling and the development of fibrotic changes. At the same time, TGF-β participates in regulating the balance between pro-inflammatory and anti-inflammatory processes, making it one of the key mediators of chronic inflammation and tissue remodeling. Under physiological conditions, TGF-β signaling plays a fundamental role in maintaining normal biological functions, including embryonic development, wound healing, tissue regeneration, regulation of cell proliferation, migration and apoptosis, as well as the preservation of tissue and immune homeostasis [39,40,41].

TGF-β1 is widely recognized as a regulator of inflammatory processes; however, its net effect on brain vasculature appears to be highly cell-specific and, in the case of pericytes, functionally ambiguous. As demonstrated in the study by Rustenhoven et al. [42], TGF-β1 modulates human brain pericytes toward a mixed inflammatory phenotype characterized by a simultaneous attenuation of chemokines and adhesion molecules, e.g., monocyte chemoattractant protein-1 (MCP-1), vascular cell adhesion protein 1 (VCAM-1), and fractalkine (CX3CL1), and induction of mediators with established roles in vascular dysfunction, including IL-6. Given the intimate structural and functional coupling between pericytes and endothelial cells, these alterations are highly relevant to blood–brain barrier (BBB) integrity and endothelial homeostasis. In this context, the observed reduction in TGF-β1 levels by both Polish and Brazilian propolis under hypoxic conditions is of particular interest (Figure 2D). The stronger effect exhibited by Polish propolis suggests a potentially higher biological activity, possibly related to its distinct polyphenolic profile. Attenuation of TGF-β1 signaling may mitigate downstream induction of IL-6, thereby potentially contributing to the preservation of BBB integrity and reduction in oxidative stress within the cerebral endothelium. EEP-BRA caused a pronounced, statistically significant increase in IL-6 concentrations (171.6 pg/mL compared with 27.6 pg/mL in the control group; *p* < 0.001) and GM-CSF (8.82 compared with 5.19 pg/mL). Despite IL-6 stimulation, EEP-BRA retained its ability to inhibit fibrosis, reducing TGF-β1 levels (1114.2 pg/mL; *p* < 0.001 compared with the control group), whilst maintaining elevated levels of IL-8 (180.65 pg/mL).

Although a decrease in TGF-β1 could theoretically diminish its inhibitory effect on leukocyte trafficking, the overall balance of evidence is consistent with a putative protective role of propolis in maintaining neurovascular function under hypoxic stress (barrier function was not assessed directly here). These findings highlight a mechanism by which propolis may exert a protective effect on blood vessels in the central nervous system and warrant further investigation into its potential application in conditions associated with BBB dysfunction.

As demonstrated in the study by Schumacher et al. [43], TGF-β signaling exerts a profound destabilizing effect on the BBB, primarily by impairing the supportive function of pericytes toward endothelial cells. In vitro, exposure to TGF-β1 and TGF-β2 resulted in a significant reduction in barrier integrity, as evidenced by decreased transendothelial electrical resistance (TEER), accompanied by a marked decline in tight-junction density and complexity in endothelial cells. These structural alterations strongly suggest that TGF-β disrupts endothelial cohesion and promotes BBB leakiness. Notably, the stronger suppressive effect of Brazilian green propolis at a concentration of 50 µg/mL on TGF-β2 levels in normoxic conditions and hypoxia conditions suggests a more pronounced capacity to modulate neurovascular homeostasis, potentially due to differences in its polyphenolic composition. By limiting TGF-β-driven metabolic and phenotypic reprogramming of pericytes, propolis may indirectly protect endothelial cells from structural and functional impairment, thereby potentially contributing to the maintenance of BBB integrity under hypoxic stress conditions. Collectively, these findings support the hypothesis that propolis exerts vasculoprotective effects in the central nervous system, at least in part through modulation of the TGF-β axis, and highlight its potential relevance in pathological states associated with BBB dysfunction.

Moreover, TGF-β is a key regulatory cytokine that exerts pleiotropic effects in glioblastoma, including the promotion of tumor invasiveness, maintenance of stem-like properties via autocrine signaling, and induction of an immunosuppressive tumor microenvironment. In the vascular compartment, TGF-β indirectly stimulates angiogenesis by inducing the expression and secretion of proangiogenic factors, including vascular endothelial growth factor (VEGF) and placental growth factor (PlGF), under both normoxic and hypoxic conditions [44,45,46]. Therefore, in this context, the observed reduction in TGF- β isoform 3 in hypoxic conditions by EEP-PL at a concentration of 10 µg/mL and EEP-BRA at a concentration of 50 µg/mL may represent a significant mechanism underlying its neurovascular protective effects.

Given the dimensionality of the dataset (eleven cytokines measured across twenty experimental conditions) and the strong intercorrelation among these mediators, per-analyte testing alone does not capture the structure of the cytokine network. The univariate analyses were therefore complemented with unsupervised multivariate exploration, namely hierarchical cluster analysis and principal component analysis, an approach previously applied to multiplex cytokine and chemokine datasets in a neuro-inflammatory context [47]. These analyses are descriptive rather than predictive: the cross-validated explained variance (Q^2^) is reported alongside R^2^ to make explicit that, at the present sample size (N = 3 per condition), the multivariate models serve to visualize co-regulation patterns and origin-dependent separation of the extracts, and are not used to support formal statistical inference.

This study has several limitations that should be considered when interpreting the findings. First, the cytokine quantification was performed at a single time-point (24 h), which precludes evaluation of temporal dynamics; future kinetic studies would clarify whether the observed responses are transient, adaptive or sustained. Second, the sample size (N = 3–4) provides power to detect large effects but limits inferential precision for moderate effects, as reflected in several wide 95% confidence intervals (e.g., TGF-β1 mean difference 95% CI −5844 to +3052 pg/mL); key findings should be confirmed in larger replication sets. Third, hCMEC/D3 is an immortalized cell line that retains many but not all properties of primary brain endothelial cells; validation in primary HBMECs and in co-culture with astrocytes/pericytes is warranted. Fourth, although both propolis extracts were chemically characterized in our previous work, batch-to-batch variability of natural products may limit reproducibility; standardization of bioactive markers (CAPE, chrysin, artepillin C) by HPLC should accompany any subsequent in vivo investigation. Finally, where Tukey HSD-adjusted *p*-values did not reach significance despite large effect sizes (e.g., TGF-β1 EEP-PL-50+IFN vs. IFN-HYP), the discrepancy reflects the high within-control variance under hypoxic conditions rather than the absence of a biological effect; the three-way ANOVA confirmed the underlying treatment-by-oxygenation interactions (Supplementary Table S3).

A further limitation concerns the scope of mechanistic validation. The measured endpoint of this study is the modulation of cytokine secretion, not barrier function. Although exposure to 1% O_2_ is an established hypoxic stimulus in hCMEC/D3 cells, in which it transiently stabilizes HIF-1α and durably stabilizes HIF-2α [48], and although a significant main effect of oxygenation across several analytes (Supplementary Table S3) confirms that the oxygenation manipulation produced systematic biological changes, hypoxic activation was not verified at the level of HIF-1α accumulation, hypoxia-responsive gene expression (e.g., VEGFA, SLC2A1/GLUT1) or lactate production. Likewise, interferon-α signaling was not confirmed by STAT1 phosphorylation, induction of interferon-stimulated genes, or adhesion-molecule (ICAM-1, VCAM-1) expression, and barrier integrity was not assessed by transendothelial electrical resistance (TEER), paracellular permeability, or the tight-junction proteins ZO-1, claudin-5 and occludin. Accordingly, the present cytokine-secretion findings should not be interpreted as direct evidence of barrier protection or as evidence of specific signaling mechanisms; confirmation of hypoxic and interferon-α-driven activation, together with direct assessment of barrier integrity, is a priority for follow-up studies in this model and in primary human brain microvascular endothelial cells and astrocyte/pericyte co-cultures.

## 4. Materials and Methods

### 4.1. The Materials

Interferon-alpha (IFN-α) 3 MIU/0.5 mL was purchased from Schering-Plough, Brinny, Ireland. The human blood–brain barrier cell line hCMEC/D3-BBB cell line was obtained from Cytion (Cytion GmbH, Heidelberg, Germany). Endothelial Cell Growth Medium (C-22011) was delivered from Lonza (Walkerville, MD, USA) and supplemented by Endothelial Cell Growth Medium-2 BulletKit (CC-3162), also delivered from Lonza. Additionally, 100 U/mL penicillin and 100 μg/mL streptomycin were purchased from PAA Laboratories (Pasching, Austria). Dimethyl sulfoxide (DMSO) and Phosphate-buffered saline (PBS), Hydrochloric acid, and Sodium hydroxide were purchased from Sigma Chemical Company (St. Louis, MO, USA). 3-(4,5-dimethyl-2-thiazyl)-2,5-diphenyl-2H-tetrazolium (MTT) was purchased from Merck (Darmstadt, Germany).

### 4.2. Preparation of EEP-PL and EEP-BRA Extracts

Samples of Polish brown propolis came from south Poland (Kamianna). The identification and quantification of phenolic compounds in Polish brown propolis extract were described previously using RP-HPLC-PDA analysis [49].

Samples of Brazilian green propolis came from southeastern Brazil (the state of Minas Gerais), where *Baccharis dracunculifolia* DC. is the predominant botanical source of this green propolis type. The raw propolis, collected by hand from beehives, was provided by Nihon Natural Foods Co., Ltd. (Nishishinjyuku, Tokyo, Japan). The identification and quantification of phenolic compounds in Brazilian green propolis extract were described previously using HPLC-DAD and UPLC-Q-TOF-MS methods [5]. The extraction protocols were described in previous publications [49,50].

### 4.3. Culture Collection

The collection was conducted on the hCMEC/D3 provided by Cytion GmbH (Heidelberg, Germany). These cells were delivered from human temporal lobe microvessels. They were immortalized by transducing primary human microvascular endothelial cells of the brain with a lentiviral vector expressing human telomerase reverse transcriptase (hTERT). The hCMEC/D3 cells were grown at 37 °C with 5% CO_2_ in monolayer cultures. Medium was enriched by Endothelial Cell Growth Medium-2 BulletKit. To 500 mL of medium was added 0.5 mL Epidermal Growth Factor Human Recombinant, 0.5mL Recombinant Long R Insulin-Like Growth Factor, 0.5 mL Endothelial Growth Factor Vascular Human, 0.5 mL rHuman Fibroblast Growth Factor B, 0.5 mL heparin, 0.5 mL hydrocortisone and 0.5 mL Ascorbic acid. Moreover, 10 mL of Fetal Bovine Serum was added to 500 mL medium. To ensure proper growth, a 1 mL mixture containing 100 U/mL penicillin and 100 μg/mL streptomycin was added to the 100 mL medium. The passages were conducted twice a week. In order to evaluate the number of cells, a Bürker counting chamber was used. The number of cells was counted according to Formula (1):

(1) $$\mathrm{Number}\;\mathrm{of}\;\mathrm{cells}\;\mathrm{in}\;1\;\mathrm{mL}\;=\frac{4\;squares\;counted\;}{2}\times 100\times 1000$$

Finally, the suspensions of cells were diluted to 100,000 cells/mL. A quantity of 200 μL cultured cells was seeded into 96-well plates. Therefore, each well contained 20,000 cells. Stimulation by IFN-α at a concentration of 100 U/mL was conducted for 2 h. Then, in the experiment, the hCMEC/D3 cell line was treated with both propolis extracts (EEP-PL and EEP-BRA) at final concentrations of 10 μg/mL or 50 μg/mL (pre-dissolved in DMSO and then dissolved in culture medium—the final concentration of DMSO was 0.1%), either alone or in combination with IFN-α.

Immediately after adding the extracts (EEP-PL and EEP-BRA) as well as EEP-PL+IFN-α and EEP-BRA+IFN-α, the plate was placed in normoxic conditions and also in hypoxic conditions using an incubator with 1% oxygen for 24 h. Hypoxia was induced by reducing environmental oxygen to 1% O_2_ rather than by a chemical mimetic such as cobalt chloride (CoCl_2_), in order to reproduce a physiological low-oxygen environment and to avoid the off-target effects and only partial overlap with the genuine hypoxic response that have been reported for chemical HIF-stabilizing agents [51]. Under comparable conditions, 1% O_2_ has been shown to transiently stabilize HIF-1α and durably stabilizes HIF-2α in the hCMEC/D3 line [50]. All experiments were performed in triplicate.

### 4.4. The Cytotoxicity Assay—MTT Assay

In this assay, 3-(4,5-dimethyl-2-thiazyl)-2,5-diphenyl-2H-tetrazolium (MTT) was used to evaluate cell viability. This method is based on reducing 3-(4,5-dimethyl-2-thiazyl)-2,5-diphenyl-2H-tetrazolium bromide, which is transferred from viable cells to a blue formazan crystal. Propolis extracts at concentrations of 10 and 50 μg/mL with or without IFN-α were added to 96 wells, such that each well contained 200 μL. Two controls were prepared for their experimental model: a control of the line (DMSO) and a control of IFN-α. After 24 h, the medium was removed, and 180 μL of fresh medium and 20 μL of MTT solution (5 mg/mL PBS) were added to each well. The cells were allowed to incubate for four hours. The supernatant was removed, and DMSO was added to each well to dissolve the formazan crystals. The medium contained no other substances. The estimation was carried out spectrophotometrically at a wavelength of 550 nm. The results, expressed in terms of absorbance, were calculated according to Formula (2):

(2) $$\%\;\mathrm{cell}\;\mathrm{viability}\;=\;\frac{sample\;absorbance\;}{absorbance\;of\;the\;control}\times 100$$

### 4.5. Multiplex Bead-Based Cytokine Assay

A multiplex assay was used for cytokine detection. Cytokines were measured in the culture supernatant. The supernatant derived from native hCMEC/D3 stimulated by IFN-α, after incubation with and without propolis extracts for 24 h, was analyzed. Bio-Plex Human cytokine Panels–Bio-Plex Pro Human Cytokine Grp I Panel 8-Plex, Cat. #M50000007A, and Bi-Plex Pro TGF-β Panel 3-Plex, Cat. #171W4001M (BIO-RAD Laboratories, Hercules, CA, USA), were used in combination with the Bio-Plex 3D Suspension Array System based on xMAP technology (Bio-Rad Laboratories Inc., Hercules, CA, USA).

Standards and a magnetic bead suspension were prepared. Subsequently, 50 µL of each standard, samples and 50 µL of bead suspension were dispensed into the wells of a 96-well microplate. The plate was incubated for 2 h on a shaker (TITRAMAX100, Heidolph Instruments GmbH & Co. KG, Schwabach, Germany) to allow binding. After incubation, the wells were washed with 100 µL of Bio-Plex Wash Buffer using an BioTek ELx50 magnetic washer (Winooski, VT, USA). Next, 50 µL of biotinylated antibody solution was added to each well.

Following this step, the wells were washed three times with 100 µL of buffer, and 50 µL of streptavidin–phycoerythrin conjugate was added to each well. The plate was incubated for 30 min and subsequently washed again with Bio-Plex Wash Buffer. Finally, 125 µL of buffer was added to resuspend the beads.

In the case of TGF-β measurements, in the first step, samples of supernatants were activated using 1N HCl and neutralized with 1.2N NaOH/0.5 M HEPES. Then, measurements were taken as above in accordance with the manufacturer’s instructions.

The assay was performed in a single well. Quantification was carried out using a Bio-Rad Bio-Plex 3D instrument, which operates on a dual-laser flow cytometry principle. The first laser (638 nm) identified the bead region (color-coded for each analyte), while the second laser (532 nm) excited the phycoerythrin reporter to measure fluorescence intensity proportional to the amount of analyte bound. Analyte concentrations were calculated by interpolation from calibration curves constructed using the corresponding standards. All experiments were performed in triplicate.

### 4.6. Statistical Analysis

Statistical analyses were performed in TIBCO Statistica 13.3 (TIBCO Software Inc., Palo Alto, CA, USA). The biological replicate constituted the unit of analysis: N = 4 for MTT viability, and N = 3 for multiplex cytokine quantification. Data are presented as mean ± SD; the significance threshold was α = 0.05, with exact two-sided *p*-values reported to three significant figures and “*p* < 0.001” used only below that bound. Cytokine concentrations were right-skewed and analyzed after log_10_ transformation; variance homogeneity was confirmed by the Brown–Forsythe modification of Levene’s test (median-centered; all 11 analytes *p* > 0.10). Treatment differences were assessed by one-way ANOVA with Tukey HSD post hoc. A parallel Welch ANOVA, free of the variance-equality assumption, yielded qualitatively identical conclusions (Supplementary Table S2). The three experimental factors, oxygenation, stimulation, and treatment, were further evaluated by three-way factorial ANOVA on log-transformed data with Type II sum-of-squares (Supplementary Table S3). Omnibus effects are reported as η^2^ = SS_effect/SS_total; for pre-specified pairwise contrasts, Hedges’ g with 95% CI (small-sample correction J = 1 − 3/(4·df − 1)) was computed from descriptive statistics, alongside the mean difference and its Welch–Satterthwaite 95% CI. Multivariate exploration used log-transformed, z-score-standardized data. Hierarchical cluster analysis applied Ward’s minimum-variance linkage with squared Euclidean distance. Principal component analysis was carried out on the standardized correlation matrix; components with eigenvalues > 1 were retained, with cumulative explained variance (R^2^_cum) and cross-validated explained variance (Q^2^_cum) both reported—the latter to flag the model as descriptive rather than predictive. Bivariate associations were quantified by Spearman’s ρ with 95% CIs derived by Fisher’s z-transformation. At N = 3–4, the design provides ~80% power to detect large effects (Cohen’s d ≥ 1.5; α = 0.05, two-sided); where the 95% CI of an effect size spanned zero, findings were treated as biologically suggestive but statistically imprecise and flagged for confirmation in larger replication sets.

## 5. Conclusions

Polish propolis extract (EEP-PL) at a high concentration of 50 µg/mL exhibited a distinct anti-inflammatory and a potential neurovascular protective effect under hypoxic conditions, as evidenced by reductions in TNF-α (substantial reduction with large effect size, Hedges’ g = −6.78, but with omnibus *p* > 0.05 due to high variance at low cytokine levels) and IL-8 (Tukey HSD *p* < 0.05) and a consistent direction of effect on TGF-β1–β3 isoforms (with three-way ANOVA confirming significant treatment main effects despite limited Tukey-adjusted pairwise significance for several contrasts at N = 3). The Polish propolis extract is characterized by strong protective potential against excessive inflammatory reactions and tissue remodeling. In contrast, the Brazilian propolis extract (EEP-BRA) at a high concentration of 50 µg/mL induced a strong immunostimulatory effect characterized by increased production of IL-6 whilst maintaining high cell viability, suggesting the activation of cellular defense mechanisms and metabolic adaptation rather than classic inhibition of inflammation. Despite this stimulatory profile, EEP-BRA also retained significant neurovascular protective activity by reducing TGF-β1 and TGF-β2 concentrations. Overall, both propolis extracts effectively counteracted the hypoxia-induced increase in pro-inflammatory and profibrotic mediators; however, they differed in their modulation of effector cytokine pathways. EEP-PL enhanced the anti-inflammatory effect, whilst EEP-BRA more strongly activated the immune response associated with enhanced defensive signaling.

From a translational perspective, the divergent immunomodulatory profiles of the two extracts point to distinct potential applications. The anti-inflammatory, TGF-β-lowering profile of Polish propolis may be relevant to conditions in which excessive cytokine signaling and fibroproliferative remodeling compromise the neurovascular unit, such as ischemic stroke and chronic neuroinflammation, whereas the immunostimulatory profile of Brazilian propolis may be of interest where controlled immune activation is desirable. Translating these observations will require confirmation in primary human brain microvascular endothelial cells and in co-culture and in vivo models, chemical standardization of the extracts against marker constituents (CAPE, chrysin, artepillin C), and dose–response and time-course characterization. Furthermore, it should be emphasized that BBB function and the molecular mechanisms associated with hypoxia- and interferon-dependent signaling were not directly evaluated in the present study and therefore require further investigation.

## Figures and Tables

**Table 1:** Effect of EEP-PL and EEP-BRA propolis extracts on endothelial cell viability (MTT assay) under normoxic and hypoxic conditions, including IFN-α stimulation. Viability values not sharing a common superscript letter (a, b) differ significantly across the 20 experimental conditions (one-way ANOVA F(19,60) = 5.84, *p* < 0.001; Tukey HSD post hoc test, *p* < 0.05; N = 4 per condition).

Sample/Treatment	Concentration [µg/mL]	Normoxia	Normoxia (Stimulation by IFN-α)	Hypoxia	Hypoxia (Stimulation by IFN-α)
Control	0	93.53 ± 9.44 ^b^	88.83 ± 11.10 ^b^	95.42 ± 6.58 ^b^	95.27 ± 4.62 ^b^
EEP-PL	10	102.82 ± 4.62 ^ab^	98.35 ± 11.10 ^b^	100.54 ± 5.11 ^b^	111.00 ± 6.27 ^a^
	50	95.21 ± 8.75 ^b^	94.82 ± 12.67 ^b^	96.12 ± 6.07 ^b^	122.95 ± 13.17 ^a^
EEP-BRA	10	105.59 ± 10.48 ^ab^	100.12 ± 11.89 ^b^	114.46 ± 10.14 ^a^	123.42 ± 3.16 ^a^
	50	106.21 ± 4.87 ^ab^	103.36 ± 7.29 ^ab^	116.40 ± 1.79 ^a^	113.12 ± 1.21 ^a^

## Data Availability

Data will be made available upon reasonable request.

## Supplementary Materials

The following supporting information can be downloaded at https://www.mdpi.com/article/10.3390/molecules31122164/s1. Table S1: Descriptive statistics for all measured analytes (mean ± SD, N = 3 for cytokines, N = 4 for MTT). Table S2: One-way ANOVA omnibus tests on log-transformed data (classical F + Welch’s robust F) for all 11 cytokines. Table S3: Three-way factorial ANOVA on log-transformed data—main effects and interactions. Table S4: Hedges’ g effect sizes with 95% confidence intervals for pre-specified pairwise contrasts. Table S5: Post-hoc Tukey HSD adjusted *p*-values for key pairwise comparisons. Figure S1: PCA scree plot with cumulative R^2^ and Q^2^ (cross-validated). Figure S2: Spearman rank correlation matrix between cytokines.

## Abbreviations

The following abbreviations are used in this manuscript:

CAPE	Caffeic acid phenethyl ester
EEP-BRA	Brazilian green propolis
EEP-PL	Polish brown propolis
hCMEC/D3	Human brain microvascular endothelial cells
IL	Interleukin
IFN-γ	Interferon gamma
IFN-α	Interferon alpha
GM-CSF	Granulocyte–macrophage colony-stimulating factor
TNF-α	Tumor necrosis factor-alpha
TGF-β	Transforming growth factor-beta
MTT	3-(4,5-dimethyl-2-thiazyl)-2,5-diphenyl-2H-tetrazolium
IFN-NORM	Interferon alfa stimulation in normoxia
IFN-HYP	Interferon alfa stimulation in hypoxia
BBB	Blood–brain barrier
BMECs	Cultured brain microvascular endothelial cells
ZO-1	Zonula occludens-1
NF-ĸB	Nuclear factor kappa-light-chain-enhancer of activated B cells
STAT3	Signal transducer and activator of transcription 3
MAPK	Mitogen-activated protein kinases
HBEC-5i	Cerebral microvascular endothelial cell line
MCP-1	Monocyte chemoattractant protein-1
COX-2	Cyclooxygenase-2
iNOS	Inducible nitric oxide synthase
HMGB1	High-mobility group box 1 protein
TLR4/MyD88	Toll-like receptor 4/myeloid differentiation primary response 88
VCAM-1	Vascular cell adhesion protein 1
CX3CL1	Fractalkine
TEER	Transendothelial electrical resistance
PlGF	Placental growth factor
HBMECs	Human brain microvascular endothelial cell primary cells
DMSO	Dimethyl sulfoxide
PBS	Phosphate-buffered saline
