# Supplementary material for: Immunomodulatory Effects of Propolis on Endothelial Cytokine Release
Source: Molecules. 2026 Jun 19;31(12):2164. doi: 10.3390/molecules31122164 (PMC13305527; doi:10.3390/molecules31122164)
Supplement: Supplementary file 1 [file molecules-31-02164-s001.zip › molecules-4365766-supplementary.pdf]

Supplementary Materials

Immunomodulatory Effects of Propolis on Endothelial Cytokine Release

Anna Kurek-Górecka [1,*], Małgorzata Kłósek [1,*], Grażyna Pietsz [1], Radosław Balwierz [2] and Zenon P. Czuba [1]

[1] Department of Microbiology and Immunology, Medical University of Silesia in Katowice, Jordana 19, 41-808 Zabrze, Poland;
[2] Institute of Chemistry, University of Opole, Oleska 48, 45-052 Opole, Poland

Contents:

Table S1. Descriptive statistics for all measured analytes (mean ± SD, N = 3 for cytokines, N = 4 for MTT)

Table S2. One-way ANOVA omnibus tests on log-transformed data (classical F + Welch's robust F) for all 11 cytokines

Table S3. Three-way factorial ANOVA on log-transformed data — main effects and interactions

Table S4. Hedges' g effect sizes with 95% confidence intervals for pre-specified pairwise contrasts

Table S5. Post-hoc Tukey HSD adjusted p-values for key pairwise comparisons

Figure S1. PCA scree plot with cumulative R^2 and Q^2 (cross-validated)

Figure S2. Spearman rank correlation matrix between cytokines

Table S1. Descriptive Statistics

Mean ± SD for each of the 20 experimental conditions (combinations of Oxygenation × Stimulation × Treatment) and for each of 11 cytokines plus MTT cell viability. N = 3 biological replicates for cytokines (multiplex bead-based immunoassay, single well per replicate); N = 4 for MTT (4 technical wells averaged before analysis). All values represent original (untransformed) data.

S1A. Selected proinflammatory cytokines (IL-6, IFN-γ, GM-CSF, TNF-α, IL-2)

Condition	IL-6	IFN-γ	GM-CSF	TNF-α	IL-2
VEH-NORM.	27.55 ± 2.18	4.74 ± 0.5	10.12 ± 3.95	5.08 ± 3.4	0.35 ± 0.34
IFN-NORM.	38.06 ± 0.95	2.79 ± 2.1	4.87 ± 2.13	3.05 ± 2.29	0.48 ± 0.3
EEP-PL-10-NORM.	46.46 ± 2.5	3.0 ± 0.51	6.54 ± 2.19	5.07 ± 1.47	0.28 ± 0.23
EEP-PL-50-NORM.	67.63 ± 7.09	0.5 ± 0.06	4.2 ± 1.54	3.22 ± 1.37	0.11 ± 0.08
EEP-PL-10-IFN-NORM.	67.44 ± 14.39	2.96 ± 2.1	7.37 ± 4.42	3.4 ± 1.1	0.28 ± 0.31
EEP-PL-50-IFN-NORM.	89.64 ± 48.75	3.22 ± 2.43	10.96 ± 5.03	3.73 ± 2.85	0.18 ± 0.15
EEP-BRA-10-NORM.	35.02 ± 1.01	4.43 ± 4.95	7.17 ± 3.94	4.07 ± 4.77	1.54 ± 1.55
EEP-BRA-50-NORM.	140.37 ± 17.25	2.37 ± 2.47	8.75 ± 2.62	3.28 ± 2.03	1.04 ± 0.62
EEP-BRA-10-IFN-NORM.	53.45 ± 0.55	3.7 ± 0.56	9.57 ± 0.97	3.58 ± 2.07	0.45 ± 0.23
EEP-BRA-50-IFN-NORM.	192.92 ± 8.32	3.37 ± 1.47	8.03 ± 1.67	4.97 ± 2.51	0.46 ± 0.51
VEH-HYP.	16.25 ± 1.44	2.73 ± 0.56	6.18 ± 1.5	3.12 ± 1.01	0.35 ± 0.04
IFN-HYP.	27.63 ± 0.76	0.73 ± 1.17	5.19 ± 1.17	3.61 ± 0.29	0.34 ± 0.03
EEP-PL-10-HYP.	35.85 ± 3.54	0.28 ± 0.02	8.39 ± 2.98	1.93 ± 1.45	0.7 ± 0.47
EEP-PL-50-HYP.	92.48 ± 10.22	3.74 ± 5.94	10.32 ± 2.11	2.05 ± 3.14	1.14 ± 1.19
EEP-PL-10-IFN-HYP.	51.33 ± 21.88	1.36 ± 1.06	8.97 ± 1.82	2.77 ± 3.14	0.35 ± 0.15
EEP-PL-50-IFN-HYP.	69.44 ± 50.91	0.65 ± 0.65	8.29 ± 1.24	0.54 ± 0.42	0.27 ± 0.14
EEP-BRA-10-HYP.	26.79 ± 3.75	3.02 ± 2.61	8.11 ± 3.72	3.21 ± 3.27	0.42 ± 0.41
EEP-BRA-50-HYP.	79.98 ± 28.39	1.24 ± 1.06	7.8 ± 1.76	1.96 ± 0.85	0.44 ± 0.23
EEP-BRA-10-IFN-HYP.	47.41 ± 16.03	1.59 ± 0.5	9.97 ± 1.56	1.74 ± 1.12	0.84 ± 0.89
EEP-BRA-50-IFN-HYP.	171.58 ± 26.48	2.04 ± 2.59	8.82 ± 1.16	5.62 ± 1.64	0.78 ± 0.7

All values: pg/mL. Cells with extremely small values (< LLOQ ≈ 0.02 pg/mL for IL-4) should be interpreted with caution.

S1B. Anti-inflammatory and regulatory cytokines (IL-4, IL-8, IL-10)

Condition	IL-4	IL-8	IL-10
VEH-NORM.	0.44 ± 0.07	270.55 ± 69.77	0.39 ± 0.06
IFN-NORM.	0.49 ± 0.17	319.71 ± 16.91	0.36 ± 0.01
EEP-PL-10-NORM.	0.39 ± 0.08	370.0 ± 76.88	0.37 ± 0.02
EEP-PL-50-NORM.	0.16 ± 0.08	83.14 ± 9.82	0.31 ± 0.04
EEP-PL-10-IFN-NORM.	0.49 ± 0.05	340.47 ± 18.09	0.37 ± 0.04
EEP-PL-50-IFN-NORM.	0.29 ± 0.12	95.5 ± 30.19	0.31 ± 0.03
EEP-BRA-10-NORM.	0.47 ± 0.23	271.08 ± 92.88	0.36 ± 0.09
EEP-BRA-50-NORM.	0.68 ± 0.05	266.47 ± 134.88	0.36 ± 0.06
EEP-BRA-10-IFN-NORM.	0.55 ± 0.25	292.91 ± 28.61	0.35 ± 0.01
EEP-BRA-50-IFN-NORM.	0.42 ± 0.19	221.13 ± 71.03	0.43 ± 0.14
VEH-HYP.	0.51 ± 0.11	145.74 ± 18.58	0.39 ± 0.05
IFN-HYP.	0.44 ± 0.32	153.49 ± 63.7	0.36 ± 0.03
EEP-PL-10-HYP.	0.09 ± 0.12	248.92 ± 175.8	0.32 ± 0.02
EEP-PL-50-HYP.	0.39 ± 0.65	67.64 ± 9.22	0.35 ± 0.06
EEP-PL-10-IFN-HYP.	0.17 ± 0.27	193.89 ± 28.13	0.26 ± 0.05
EEP-PL-50-IFN-HYP.	0.02 ± 0.0	69.1 ± 12.21	0.28 ± 0.04
EEP-BRA-10-HYP.	0.61 ± 0.31	191.93 ± 40.32	0.31 ± 0.01
EEP-BRA-50-HYP.	0.45 ± 0.2	182.73 ± 54.43	0.33 ± 0.02
EEP-BRA-10-IFN-HYP.	0.5 ± 0.31	201.47 ± 71.64	0.46 ± 0.13
EEP-BRA-50-IFN-HYP.	0.64 ± 0.2	180.65 ± 31.6	0.34 ± 0.04

S1C. TGF-β isoforms (TGF-β1, TGF-β2, TGF-β3)

Condition	TGF-β1	TGF-β2	TGF-β3
VEH-NORM.	2939.94 ± 1230.64	1249.11 ± 258.87	67.54 ± 68.5
IFN-NORM.	3679.54 ± 734.02	1377.46 ± 133.05	105.67 ± 16.05
EEP-PL-10-NORM.	2639.1 ± 338.59	1022.95 ± 195.85	37.3 ± 20.98
EEP-PL-50-NORM.	2878.96 ± 496.59	1221.92 ± 186.39	93.09 ± 33.95
EEP-PL-10-IFN-NORM.	4357.18 ± 823.07	1318.85 ± 115.5	113.62 ± 32.34
EEP-PL-50-IFN-NORM.	2187.99 ± 566.73	793.3 ± 136.69	77.58 ± 16.75
EEP-BRA-10-NORM.	3196.92 ± 918.18	1005.85 ± 446.39	142.83 ± 64.98
EEP-BRA-50-NORM.	1332.57 ± 382.39	221.71 ± 198.02	16.6 ± 16.91
EEP-BRA-10-IFN-NORM.	3514.11 ± 136.53	660.33 ± 311.76	113.84 ± 7.07
EEP-BRA-50-IFN-NORM.	1287.82 ± 562.7	412.42 ± 274.02	59.72 ± 38.58
VEH-HYP.	1199.86 ± 1796.68	315.59 ± 202.61	21.55 ± 37.12
IFN-HYP.	2124.64 ± 1802.95	1214.01 ± 713.11	54.11 ± 92.34
EEP-PL-10-HYP.	231.71 ± 207.15	454.45 ± 60.57	8.3 ± 14.17
EEP-PL-50-HYP.	401.97 ± 298.03	686.03 ± 615.71	20.08 ± 34.57
EEP-PL-10-IFN-HYP.	458.63 ± 491.02	528.74 ± 145.45	41.51 ± 36.26
EEP-PL-50-IFN-HYP.	728.64 ± 126.94	688.36 ± 142.11	50.76 ± 31.94
EEP-BRA-10-HYP.	1418.07 ± 503.18	504.23 ± 392.26	66.72 ± 56.87
EEP-BRA-50-HYP.	554.96 ± 679.82	155.93 ± 117.48	18.45 ± 29.05
EEP-BRA-10-IFN-HYP.	2694.48 ± 517.79	797.69 ± 256.06	136.82 ± 78.26
EEP-BRA-50-IFN-HYP.	1114.21 ± 641.05	295.02 ± 205.34	81.73 ± 46.86

S1D. Cell viability (MTT assay)

Condition	Cell viability [%], mean ± SD (N=4)
VEH-NORM.	93.5 ± 9.4
IFN-NORM.	88.8 ± 11.1
EEP-PL-10-NORM.	102.8 ± 4.6
EEP-PL-50-NORM.	95.2 ± 8.8
EEP-PL-10-IFN-NORM.	98.4 ± 11.1
EEP-PL-50-IFN-NORM.	94.8 ± 12.7
EEP-BRA-10-NORM.	105.6 ± 10.5
EEP-BRA-50-NORM.	106.2 ± 4.9
EEP-BRA-10-IFN-NORM.	100.1 ± 11.9
EEP-BRA-50-IFN-NORM.	103.4 ± 7.3
VEH-HYP.	95.4 ± 6.6
IFN-HYP.	95.3 ± 4.6
EEP-PL-10-HYP.	100.5 ± 5.1
EEP-PL-50-HYP.	96.1 ± 6.1
EEP-PL-10-IFN-HYP.	111.0 ± 6.3
EEP-PL-50-IFN-HYP.	122.9 ± 13.2
EEP-BRA-10-HYP.	114.5 ± 10.1
EEP-BRA-50-HYP.	116.4 ± 1.8
EEP-BRA-10-IFN-HYP.	123.4 ± 3.2
EEP-BRA-50-IFN-HYP.	113.1 ± 1.2

ISO 10993-5:2009 cytotoxicity threshold: cell viability < 70% indicates cytotoxic concentration. All tested concentrations of EEP-PL and EEP-BRA preserved cell viability above this threshold under both normoxic and hypoxic conditions.

Table S2. One-way ANOVA Results

Classical one-way ANOVA and Welch's F-test on log-transformed cytokine concentrations and MTT cell viability. The grouping factor is the experimental condition ('Sample code FULL', 20 levels). Welch's test is provided as a sensitivity check that does not assume variance homogeneity. Bold p-values indicate statistical significance at $\alpha = 0.05$.

Variable	SS Effect	df Eff.	MS Effect	SS Error	df Err.	F	p	η^2	Welch F	Welch df_2	Welch p
log_IL-6	4.479	19	0.236	0.465	40	20.28	<0.001	0.906	187.85	14.44	<0.001
log_IFN-γ	8.760	19	0.461	14.488	40	1.27	0.260	0.377	1.45	14.49	0.239
log_GM-CSF	0.793	19	0.042	0.967	40	1.73	0.072	0.451	0.75	14.69	0.725
log_TNF-α	4.377	19	0.230	5.526	40	1.67	0.086	0.442	1.65	14.38	0.170
log_IL-2	5.072	19	0.267	8.625	40	1.23	0.278	0.370	0.75	14.46	0.725
log_IL-4	11.690	19	0.615	5.645	40	4.36	<0.001	0.674	136.44	14.60	<0.001
log_IL-8	2.803	19	0.148	0.683	40	8.64	<0.001	0.804	27.98	14.62	<0.001
log_IL-10	0.186	19	0.010	0.167	40	2.34	0.012	0.527	3.31	14.61	0.015
log_TGF-β1	10.980	19	0.578	4.498	40	5.14	<0.001	0.709	11.42	14.36	<0.001
log_TGF-β2	5.592	19	0.294	2.562	40	4.59	<0.001	0.686	7.56	14.65	<0.001
log_TGF-β3	36.327	19	1.912	36.510	40	2.09	0.024	0.499	1.78	14.37	0.135

Notes: η^2 = SS_effect / (SS_effect + SS_error); Cohen's interpretation: $\eta^2 > 0.14$ = large effect. Welch df_2 = Welch-Satterthwaite degrees of freedom (denominator). Computed in TIBCO Statistica 13.3.

Table S3. Three-way Factorial ANOVA

Univariate three-way factorial ANOVA on log-transformed data with Type II decomposition for the four target analytes that are central to the manuscript narrative (TGF-β1, TGF-β2, TGF-β3, TNF-α). Factors: Oxygenation (NORM vs HYP), Stimulation (NONE vs IFN-α), Treatment (5 levels: VEH, EEP-PL-10, EEP-PL-50, EEP-BRA-10, EEP-BRA-50). Sigma-restricted parameterisation, effective hypothesis decomposition. Computed in TIBCO Statistica 13.3.

Effect	df	log_TGF-β1 F	p	log_TGF-β2 F	p	log_TGF-β3 F	p	log_TNF-α F	p
Oxygenation	1	51.78	<0.001	15.08	<0.001	10.25	0.003	8.83	0.005
Stimulation	1	6.81	0.013	5.60	0.023	9.45	0.004	0.01	0.930
Treatment	4	4.52	0.004	12.70	<0.001	2.39	0.067	2.54	0.055
Oxygenation × Stimulation	1	4.35	0.043	3.90	0.055	1.05	0.311	0.17	0.686
Oxygenation × Treatment	4	3.55	0.014	0.67	0.617	1.37	0.262	2.00	0.113
Stimulation × Treatment	4	0.22	0.926	1.43	0.241	0.53	0.717	0.73	0.577
Oxygenation × Stim × Treat	4	0.39	0.817	0.89	0.482	0.48	0.752	0.40	0.806
Error	40	—	—	—	—	—	—	—	—

Note: Significant main effects of Oxygenation, Stimulation, and Treatment for TGF-β1 confirm that propolis treatment exerts a measurable effect across the experimental design, even when Tukey-adjusted pairwise comparisons did not reach significance under conditions of high within-group variance (e.g., IFN-HYP control with SD ≈ 1803 pg/mL).

Table S4. Effect Sizes (Hedges' g) for Pre-Specified Contrasts

Hedges' g effect sizes with 95% confidence intervals for the 10 pre-specified pairwise contrasts highlighted in the Results section. Effect sizes were computed on raw (untransformed) data using the standard formula: $g = J \times (M_1 - M_2) / s\_pool$, where s_pool is the pooled standard deviation and $J = 1 - 3/(4 \cdot df - 1)$ is the Hedges-Olkin small-sample correction. 95% confidence intervals were calculated as $g \pm 1.96 \times SE\_g$. Cohen's interpretation: $|g| < 0.5$ small, 0.5–0.8 medium, > 0.8 large, > 1.5 very large.

Comparison	Cytokine	$M_1 \pm SD_1$	$M_2 \pm SD_2$	Hedges' g	95% CI	Interpretation
EEP-PL-50+IFN vs IFN (HYP)	TGF-β1	728.6 ± 126.9	2124.6 ± 1803.0	−0.87	[−2.58, +0.84]	Large
EEP-BRA-50+IFN vs IFN (HYP)	TGF-β1	1114.2 ± 641.1	2124.6 ± 1803.0	−0.60	[−2.25, +1.06]	Medium
EEP-BRA-50 vs VEH (NORM)	TGF-β2	221.7 ± 198.0	1249.1 ± 258.9	−3.57	[−6.51, −0.62]	Very large
EEP-BRA-50 vs VEH (NORM)	TGF-β3	16.6 ± 16.9	67.5 ± 68.5	−0.82	[−2.51, +0.87]	Large
EEP-PL-50+IFN vs IFN (HYP)	TNF-α	0.54 ± 0.42	3.61 ± 0.29	−6.78	[−11.79, −1.83]	Very large
EEP-PL-50 vs VEH (NORM)	IL-8	83.1 ± 9.8	270.5 ± 69.8	−3.01	[−5.64, −0.38]	Very large
EEP-PL-50+IFN vs IFN (NORM)	IL-8	95.5 ± 30.2	319.7 ± 16.9	−7.33	[−12.66, −2.00]	Very large
EEP-PL-50 vs VEH (NORM)	IL-6	67.6 ± 7.1	27.6 ± 2.2	+6.11	[+1.58, +10.64]	Very large
EEP-BRA-50 vs VEH (NORM)	IL-6	140.4 ± 17.3	27.6 ± 2.2	+7.34	[+2.01, +12.67]	Very large
EEP-BRA-50+IFN vs VEH (NORM)	IL-6	192.9 ± 8.3	27.6 ± 2.2	+21.76	[+6.59, +36.92]	Very large

Where 95% CI of Hedges' g spans zero (e.g., TGF-β1 EEP-PL-50+IFN vs IFN-HYP), the effect estimate is statistically imprecise at N = 3 due to high within-group variance of the IFN-HYP control (CV ≈ 85%). The biological direction of effect remains consistent with the three-way ANOVA main effects of Treatment (Table S3).

Table S5. Tukey HSD Adjusted p-values — Key Pairwise Comparisons

Tukey honestly significant difference (HSD) post-hoc test on log-transformed data for the most relevant pairwise contrasts (treatment vs. corresponding vehicle/IFN control under the same oxygenation × stimulation conditions). Computed in TIBCO Statistica 13.3.

Cytokine	Treatment vs. Control (under matching conditions)	Tukey HSD p	Significance
IL-6	EEP-PL-50 vs VEH (NORM)	0.009	**
IL-6	EEP-BRA-50 vs VEH (NORM)	<0.001	***
IL-6	EEP-BRA-50+IFN vs VEH (NORM)	<0.001	***
IL-6	EEP-BRA-50+IFN vs IFN (HYP)	<0.001	***
IL-8	EEP-PL-50 vs VEH (NORM)	0.004	**
IL-8	EEP-PL-50+IFN vs IFN (NORM)	0.002	**
TGF-β1	EEP-PL-50+IFN vs IFN (HYP)	0.997	n.s.
TGF-β1	EEP-BRA-50+IFN vs IFN (HYP)	1.00	n.s.
TGF-β2	EEP-BRA-50 vs VEH (NORM)	0.013	*
TGF-β2	EEP-BRA-50 vs VEH (HYP)	0.911	n.s.
TGF-β2	EEP-BRA-50+IFN vs IFN (HYP)	0.219	n.s.
TGF-β3	EEP-BRA-50 vs VEH (NORM)	1.00	n.s.
TGF-β3	EEP-BRA-50 vs VEH (HYP)	1.00	n.s.
TNF-α	EEP-PL-50+IFN vs IFN (HYP)	0.297	n.s.

*Significance code: *** $p < 0.001$, ** $p < 0.01$, * $p < 0.05$, n.s. = not significant. Note: Several biologically meaningful contrasts (e.g., TGF-β1 EEP-PL-50+IFN vs IFN-HYP, TNF-α EEP-PL-50+IFN vs IFN-HYP) did not reach significance in pairwise testing despite very large effect sizes ($|g| > 0.8$). This reflects the high within-group variance of hypoxic IFN-α controls combined with the multiplicity correction across 190 pairwise comparisons. The three-way ANOVA (Table S3) confirms the underlying significant main effects of Treatment for TGF-β1 ($p = 0.004$) and main effect of Oxygenation for TNF-α ($p = 0.005$).*

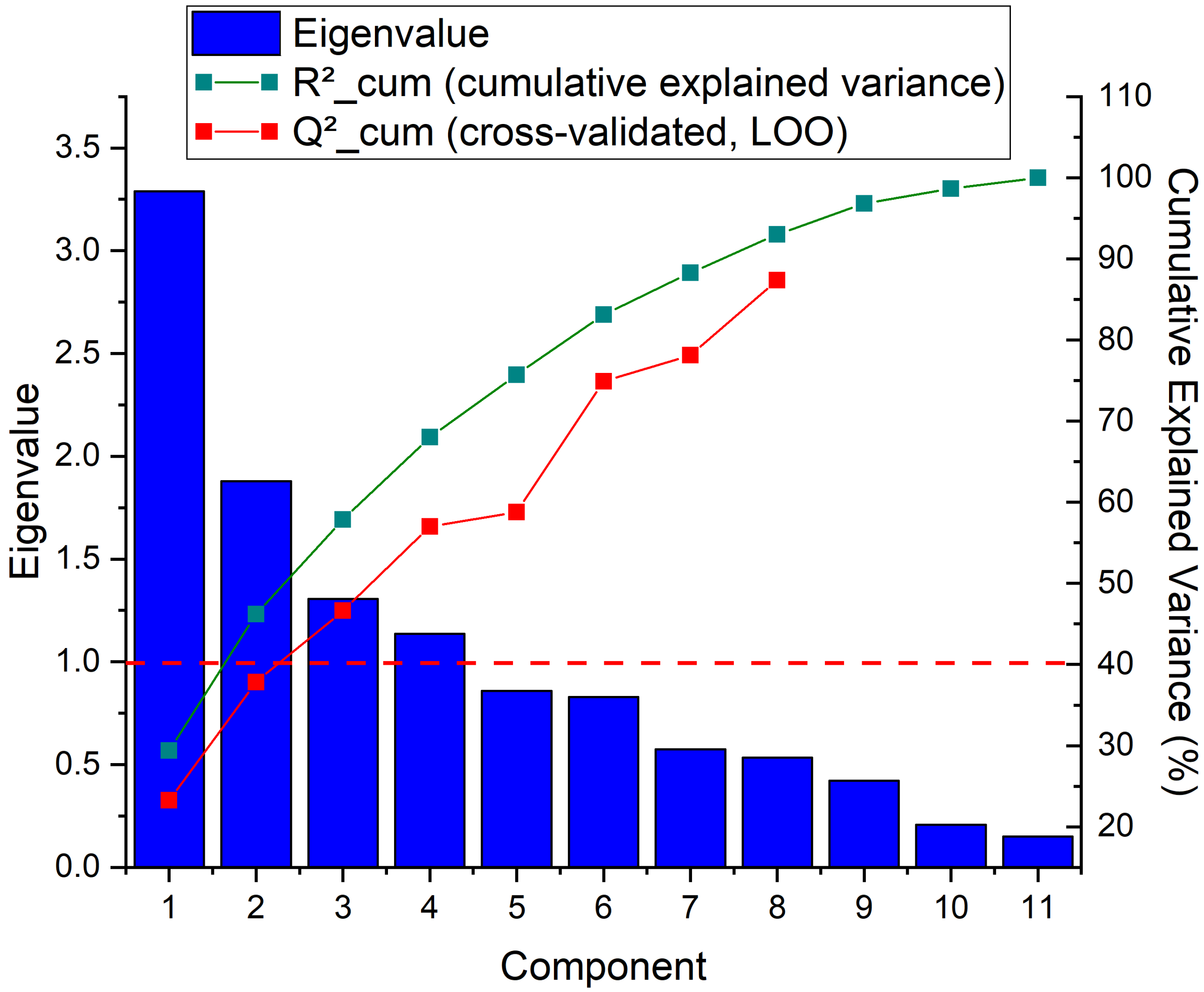

Figure S1. PCA scree plot with cumulative R^2 and Q^2 (cross-validated)

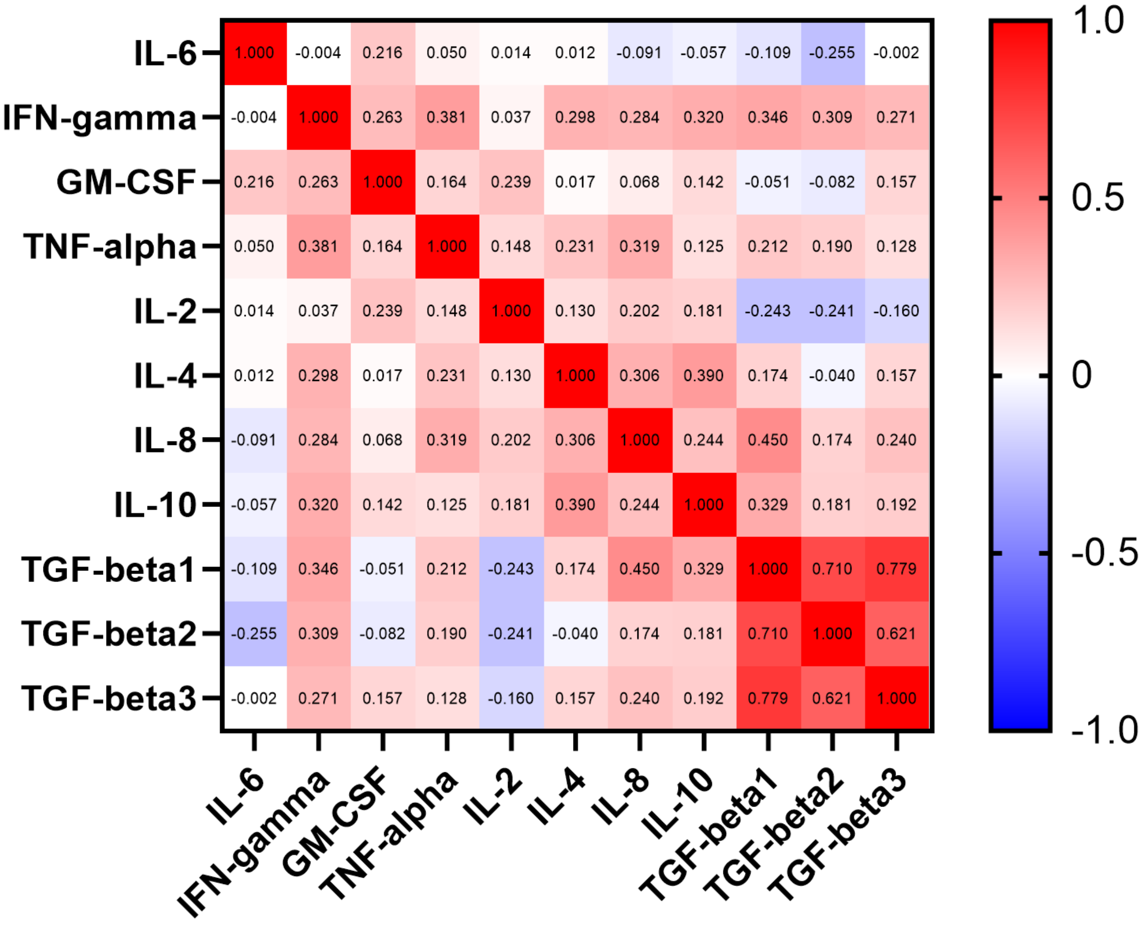

Figure S2. Spearman rank correlation matrix between cytokines
